# Codon Usage Bias Analysis of Bluetongue Virus Causing Livestock Infection

**DOI:** 10.3389/fmicb.2020.00655

**Published:** 2020-05-19

**Authors:** Xiaoting Yao, Qinlei Fan, Bo Yao, Ping Lu, Siddiq Ur Rahman, Dekun Chen, Shiheng Tao

**Affiliations:** ^1^State Key Laboratory of Crop Stress Biology in Arid Areas, College of Life Sciences, Northwest A&F University, Yangling, China; ^2^College of Veterinary Medicine, Northwest A&F University, Yangling, China; ^3^China Animal Health and Epidemiology Center, Qingdao, China; ^4^Department of Computer Science and Bioinformatics, Khushal Khan Khattak University, Karak, Pakistan

**Keywords:** bluetongue virus, *Reoviridae*, *Culicoides*, nucleotide composition, codon usage bias, evolution

## Abstract

Bluetongue virus (BTV) is a double-stranded RNA virus with multiple segments and belongs to the genus *Orbivirus* within the family *Reoviridae*. BTV is spread to livestock through its dominant vector, biting midges of genus *Culicoides*. Although great progress has been made in genomic analyses, it is not fully understood how BTVs adapt to their hosts and evade the host’s immune systems. In this study, we retrieved BTV genome sequences from the National Center for Biotechnology Information (NCBI) database and performed a comprehensive research to explore the codon usage patterns in 50 BTV strains. We used bioinformatic approaches to calculate the relative synonymous codon usage (RSCU), codon adaptation index (CAI), effective number of codons (ENC), and other indices. The results indicated that most of the overpreferred codons had A-endings, which revealed that mutational pressure was the major force shaping codon usage patterns in BTV. However, the influence of natural selection and geographical factors cannot be ignored on viral codon usage bias. Based on the RSCU values, we performed a comparative analysis between BTVs and their hosts, suggesting that BTVs were inclined to evolve their codon usage patterns that were comparable to those of their hosts. Such findings will be conducive to understanding the elements that contribute to viral evolution and adaptation to hosts.

## Introduction

Bluetongue virus (BTV) causes a vector-borne viral disease [bluetongue (BT)], is an economically important virus of ruminants that belongs to the genus *Orbivirus* of the *Reoviridae* family, and has a genome that consists of multiple segments of double-stranded RNA. Some infected animals develop the disease known as BT, with reference to the characteristic cyanotic tongue and lip mucosa (Saegerman et al., 2007; Maclachlan et al., 2009). According to electrophoretic analyses, BTV proteins are divided into a large fragment group, medium fragment group, and small fragment group (Rossmann and Tao, 1999; Diprose et al., 2001, 2002). BTV is transmitted to animals through its primary vector, biting midges of genus *Culicoides*, however, it is also transmitted directly through the placenta or by sex (De Clercq et al., 2008; Courtejoie et al., 2018b). At present, it is clear that there are 28 serotypes of BTV (Bumbarov et al., 2020), which have been distributed worldwide, and this vector-borne viral disease is listed by the World Organization for Animal Health as an infectious disease.

Bluetongue is a viral disease that causes mild fever or facial edema in domestic ruminants and wild ungulates; livestock can die from BTV infection (Courtejoie et al., 2018b). BTV is widely distributed and has caused serious losses to countries worldwide. It was first reported in South Africa and later named by Huntcheon (Mehlhorn et al., 2007). From 1956, BTV-10 from North Africa entered Portugal and Spain, and it gave rise to the deaths of nearly 180,000 sheep; then, BTV-4 entered the Greek Islands in the period from 1979 to 1980 (Mellor et al., 2008). In 1995, by comparing the L1 gene sequences of five serotypes (BTV1, 10, 11, 13, and 17), studies showed that the nucleotide sequences of BTV1, 11, 13, and 17 were shorter than those of BTV10 (Huang et al., 1995). Phylogenetic analysis revealed that the L1 gene was the most conserved and highly homologous among the 10 gene fragments (Huang et al., 1995). Through analysis and comparison of capsid and outer coat protein nucleotide sequences, it is possible to explore the phylogenetic relationship of BTV serotypes using VP2 genes as a determinant (Gould and Pritchard, 1990). In 1999, sequence and phylogenetic analyses of the VP2 gene of BTV strains from China, Australia, South Africa, and the United States indicated that these viruses were grouped on the basis of serotype (Bonneau et al., 1999). During the years 1998–2005, BTV entered many countries that had never encountered this virus, especially around the Mediterranean basin (Purse et al., 2005). Meanwhile, there was a large pool of various BTV serotypes in Europe because of the incursions of BTV-1, BTV-2, BTV-3, BTV-4, BTV-6, BTV-9, BTV-13, and BTV-16, which constituted serious threats to mammals in Europe (Mellor et al., 2008). In 2006, BTV-8 first entered northern Europe, but the origin of the new serotype BTV-8 is still unclear (Courtejoie et al., 2018a). Most recently, a number of other new strains of BTVs have been detected that potentially signified additional virus serotypes (Savini et al., 2017). To date, some studies have implications for BTV vaccine control strategies (Batten et al., 2013; Courtejoie et al., 2018a). The live attenuated vaccines were available for many years, but were less used later with their potential safety issues (Veronesi et al., 2005). Then, the inactivated vaccines were developed and have shown great safety and efficacy in sheep and cattle (Eschbaumer et al., 2009; Gethmann et al., 2009). Currently, with the development of recombinant DNA technology, the intrinsically safe vaccines have been available and been still under development (Calvo-Pinilla et al., 2014; Marin-Lopez and Ortego, 2016). Although multiple BTV vaccines could limit the severity of viral infection, they could not completely prevent the disease.

Degeneracy of genetic codons provides a chance for evolution to improve translation efficiency while keeping the identical amino acid sequence (Chen H. et al., 2014). After a long period of evolution, the synonymous codons used by different species in the process of translation are very different (Nasrullah et al., 2015; Rahman et al., 2018). In general, 64 codons encode 20 various amino acids and three termination codons; therefore, most of the codons are synonymous in the translation process (Chen H. et al., 2014). Notably, synonymous codons appear with different frequencies while coding for the same amino acid, which is known as codon usage bias (Ikemura, 1981, 1985). The investigation of molecular evolution shows that codon usage bias is widespread in viruses, prokaryotes, and eukaryotes and even exists among different genes in the same organism (Greenbaum et al., 2008; Butt et al., 2016; Rahman et al., 2018). Codon preference is more obvious in genes with higher expression levels than in those with lower expression levels (Jia and Higgs, 2008), which may be caused by mutational and selection forces (Sharp et al., 1993; Chen H. et al., 2014). Studies on codon usage have suggested that there are several factors forcing codon usage patterns, such as gene expression level, translation, protein secondary motifs, GC content, and transcriptional factors, among others (Cristina et al., 2015; Wang et al., 2016; Ur Rahman et al., 2017; Rahman et al., 2018). However, the major factors are mutational pressure and natural selection, which are thought to cause codon usage variation in organisms (Michael, 1988; Sharp et al., 2005; Chen H. et al., 2014; Butt et al., 2016).

A number of studies have suggested that when compared with natural selection, mutational pressure is the major force establishing codon usage patterns (Sharp et al., 2010; Cristina et al., 2015). However, mutational pressure is not the only driving factor for various DNA or RNA viruses (Butt et al., 2014; Rahman et al., 2018). Compared with the genomes of prokaryotes and eukaryotes, there are some specific features in viral genomes, for instance, depending on their hosts to replicate, synthesize, and transmit protein. This interaction between virus and host is thought to influence the viral survival, adaptation, evolution, and immune escape from the host’s immune system (Shackelton et al., 2006; Moratorio et al., 2013; Butt et al., 2014; Rahman et al., 2018). Accordingly, understanding of codon usage in viral genomes may improve the knowledge of molecular evolution and enhance our insight into the regulation of viral gene expression (Butt et al., 2014; Rahman et al., 2018). Thus, the codon usage pattern is a vital element to reflect the evolutionary process and BTV molecular mechanism in escaping host cell responses.

This study focused on 50 different strains of BTV and performed viral genomic analyses for codon usage patterns using available sequences data. We found that mutational pressure makes an important impact on building codon usage patterns in BTV genomes.

## Materials and Methods

### Data Description

In our research, complete genomic sequences of 50 BTVs were retrieved from the National Center for Biotechnology Information (NCBI)^1^. Supplementary Table S2 shows the sequence information. For each strain, the ORFs were obtained by Lasergene SeqBuilder (Singh et al., 2016) and aligned using the MUSCLE program (Goñi et al., 2012). Additionally, codon usage data of BTV’s hosts, *B. taurus*, *O. aries*, and *Culicoides*, were acquired from the codon usage database^2^.

### Nucleotide Components Analysis

Nucleotide compositional analysis of the 50 BTV genomic sequences was analyzed by online software, CAIcal^3^, and local software, codonW^4^. The whole nucleotide frequencies of four types of nucleotides that occurred at the third codon position (U3, G3, C3, and A3) and G + C nucleotides that occurred at the first (GC1), second (GC2), and third (GC3) positions were calculated. In addition, the mean frequency of GC at the first two positions (GC12) and the ratio of AU/CG were also calculated. In this study, we excluded the three stop codons (UAA, UAG, and UGA), AUG and UGG (no synonymous codon).

### Codon Preference Characteristics

To determine the codon usage bias pattern of BTV coding sequences, the relative synonymous codon usage (RSCU) of the virus genome coding region was calculated by the software codonW (Sharp and Li, 1986), and dinucleotide content was calculated by SSE v1.2 editor software (Karniychuk, 2016). Furthermore, another vital index of the codon usage pattern is the effective number of codons (ENC). The formula is as follows:

$$\mathrm{ENC}=2+\frac{9}{F_{2}}+\frac{1}{F_{3}}+\frac{5}{F_{4}}+\frac{3}{F_{6}}$$

$$F=\frac{n\,\sum_{i=1}^{k}p_{i}^{2}-1}{n-1}\;n>1\;p_{i}=\frac{n_{i}}{n}$$

where *F*_*i*_ is the average homozygosity evaluated for synonymous family type *i*; *n* indicates the number of codons in the sequences; *k* indicates the types of synonymous codons that encode the same amino acid; and *p*_*i*_ indicates the ratio of the *i* codon to all codon numbers encoding the same amino acid (Wright, 1990).

### ENC-Plot Analysis

An ENC plot can clarify the relationship between the ENC and the GC content at the third codon position (GC3). This method can vividly demonstrate the usage bias of gene codons. To evaluate the correlation, the expected ENC values were calculated for the corresponding GC3 using the method of Singh et al. (2016):

$$E\,N\,C^{\exp e\,c\,t\,e\,d}=2+s+\left(\frac{29}{s^{2}+{\left(1-S\right)}^{2}}\right)$$

where *s* represents G + C contents at the third codon position (GC3s).

### Neutral Evolution Analysis

Neutral evolution analysis or the neutrality plot analysis is used to determine the factors that influence the preference of codon usage (Nasrullah et al., 2015). This analysis was performed to determine and compare the extent of influence of mutation pressure and natural selection on the codon usage patterns of BTV by plotting the GC12 values of the synonymous codons against the GC3 values.

### Correspondence Analysis (COA)

Correspondence analysis is a multivariate statistical analysis that is used to detect variable and sample relationships. COA displays sets of rows and columns in a particular data set (Wong et al., 2010). In this study, every ORF corresponds to 59 dimensions (59 codons) and every dimension is equivalent to the RSCU value for each codon (except for the Met, Tyr, and stop codons). This approach helps to reflect directly the trend of strain change. The codonW program was used to perform COA based on the RSCU values, and the R ggplot2 package was used to draw visual graphics.

### Correlation Analysis

Correlation analysis was used to measure the correlation between variables. Spearman’s rank correlation method was performed to analyze the relationship between the codon usage pattern and nucleotide content of the BTV genome (Wu et al., 2015). All statistical procedures were carried out using the R corrplot package, and the related indicators of codon usage bias were obtained by using codonW.

## Results

### Nucleotide Contents Analysis in BTV

**FIGURE 1:**
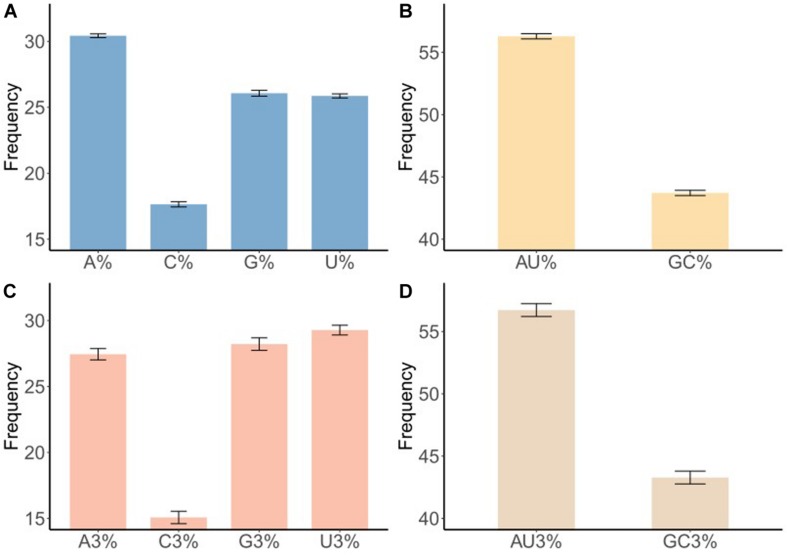
Nucleotide content distribution and composition. **(A)** The mean frequency for A, U, G, and C composition in 50 different BTV sequences are shown. **(B)** The mean frequency for AU and GC composition indicates AU richness. **(C)** The mean values of the nucleotide content frequency at the third codon position. **(D)** Analysis for AU and GC composition at the third codon position suggests higher AU content than GC at the third codon position. Standard deviation was marked in the plot.

Codon usage patterns are considered to be largely affected by the nucleotide composition (Jenkins and Holmes, 2003; Wong et al., 2010). The nucleotide contents of the BTV complete coding sequences were measured to evaluate the impact of nucleotide composition on the codon usage pattern. The frequency of each nucleotide was as follows: A (30.42% ± 0.14), U (25.86% ± 0.15), C (17.65% ± 0.20), and G (26.07% ±0.23) (Figure 1A and Table 1, wilcox.test, *P* < 0.01). It may indicate that A nucleotides of the BTV codons might be used more frequently. To further explore the nucleotide composition analysis of BTVs, mean values were considered for each codon at the third position of synonymous codons (A3, U3, G3, and C3). The percentages of nucleotide composition at the third codon position were A3 (27.45%), U3 (29.27%), G3 (28.21%), and C3 (15.07%) (Figure 1C and Table 1, wilcox.test, *P* < 0.01). The average AU and GC contents were calculated to be 56.29 and 43.71%, respectively, emphasizing that the content of AU was enriched in the BTV coding sequences (wilcox.test, *P* < 0.01). Moreover, the scope of AU3 values ranged from 53.62 to 55.63%, and the average value was 54.90%, with a standard deviation (SD) of 0.40%. GC nucleotide content at different codon positions is a significant index to show base composition bias. The scopes of GC composition are as follows: 50.20 to 51.10% (mean = 50.69%, SD = 0.22%) at the first position of all codons; 36.80 to 37.60% (mean = 37.18%, SD = 0.22%) at the second position of all codons; and 43.60 to 44.30% (mean = 43.93%, SD = 0.17%) at the first and second positions of all codons. In addition, we also calculated the mean AU (56.28% ± 0.21%), GC (43.72% ± 0.21%), AU3 (56.72% ± 0.51%), and GC3 (43.28% ± 0.51%) contents (Table 1 and Figures 1B,D), showing that A/U nucleotides are preferred at the third codon position. It is indicated that in BTV genomes, the compositional constraint plays a vital key in the total nucleotide compositions and the nucleotide composition at the third codon position.

**TABLE 1:** Nucleotide composition analysis of BTV coding sequences (%).

Sequence/parameters	A	U	G	C	GC	AU	GC1	GC2	GC12	A3	U3	G3	C3	GC3	AU3	Gravy	ARO
MG206077.1-MG206086.1	30.55	25.88	25.73	17.85	43.57	56.43	50.29	37.38	43.84	27.81	29.15	27.49	15.55	43.04	56.96	–0.34	0.09
KP339154.1-KP339163.1	30.40	26.07	25.85	17.68	43.53	56.47	50.52	37.13	43.82	27.35	29.69	27.58	15.37	42.95	57.05	–0.34	0.09
KP339234.1-KP339243.1	30.51	25.83	25.84	17.82	43.66	56.34	50.47	37.39	43.93	27.64	29.26	27.85	15.25	43.10	56.90	–0.34	0.09
KP339224.1-KP339233.1	30.38	26.03	26.03	17.56	43.59	56.41	50.54	36.82	43.68	26.95	29.64	28.38	15.04	43.42	56.58	–0.35	0.09
KP339164.1-KP339173.1	30.23	26.15	26.06	17.56	43.61	56.39	50.46	37.06	43.76	26.74	29.94	28.33	14.99	43.32	56.68	–0.34	0.09
KX599359.1-KX599368.1	30.62	25.97	25.96	17.44	43.41	56.59	50.64	36.97	43.81	27.97	29.43	28.11	14.49	42.60	57.40	–0.33	0.09
KX164149.1-KX164158.1	30.62	25.73	25.95	17.69	43.65	56.35	50.29	36.92	43.61	27.56	28.72	28.20	15.53	43.72	56.28	–0.34	0.09
KX164129.1-KX164138.1	30.23	25.94	26.40	17.43	43.83	56.17	50.89	37.25	44.07	27.16	29.48	28.89	14.47	43.36	56.64	0.33	0.09
KX164109.1-KX164118.1	30.31	25.86	26.20	17.64	43.83	56.17	50.66	37.18	43.92	27.25	29.09	28.70	14.97	43.67	56.33	–0.32	0.09
KX164099.1-KX164108.1	30.35	25.96	25.89	17.80	43.69	56.31	50.68	36.97	43.82	27.20	29.38	28.03	15.40	43.43	56.57	0.35	0.09
KX164089.1-KX164098.1	30.45	25.72	26.36	17.48	43.84	56.16	50.53	37.55	44.04	27.66	28.90	28.43	15.01	43.44	56.56	0.33	0.09
KX164079.1-KX164088.1	30.31	25.43	26.48	17.78	44.27	55.73	50.68	37.60	44.14	27.25	28.22	28.77	15.76	44.53	55.47	–0.33	0.09
KX164069.1-KX164078.1	30.41	25.83	26.02	17.74	43.76	56.24	50.77	37.40	44.09	27.67	29.22	28.01	15.10	43.11	56.89	–0.33	0.09
KX164049.1-KX164058.1	30.63	25.89	25.81	17.68	43.48	56.52	50.69	36.84	43.77	28.24	28.84	27.68	15.24	42.92	57.08	0.34	0.09
JX003687.1-JX003696.1	30.45	26.03	26.01	17.51	43.52	56.48	50.18	37.18	43.68	27.15	29.64	28.30	14.91	43.21	56.79	0.35	0.09
KU760997.1-KU761006.1	30.53	26.17	26.24	17.05	43.29	56.71	50.59	37.59	44.09	28.44	29.86	28.15	13.55	41.70	58.30	–0.31	0.09
KU760987.1-KU760996.1	30.33	26.21	26.47	16.99	43.46	56.54	50.51	37.51	44.01	27.97	29.67	28.77	13.59	42.36	57.64	0.31	0.09
KT002578.1-KT002587.1	30.26	25.84	26.27	17.63	43.90	56.10	50.51	37.28	43.89	27.09	28.98	28.71	15.22	43.92	56.08	–0.34	0.09
JX399148.1-JX399157.1	30.58	25.81	25.77	17.84	43.61	56.39	50.75	37.07	43.91	27.72	29.27	27.61	15.41	43.01	56.99	–0.35	0.09
KY654328.1-KY654337.1	30.19	25.86	26.28	17.68	43.96	56.04	50.85	37.25	44.05	26.96	29.26	28.82	14.95	43.77	56.23	–0.33	0.09
KY049853.1-KY049862.1	30.69	25.79	25.98	17.55	43.53	56.47	50.72	37.11	43.92	28.21	29.04	27.85	14.89	42.74	57.26	–0.33	0.09
KY049843.1-KY049852.1	30.75	25.88	25.97	17.40	43.37	56.63	50.72	37.07	43.90	28.46	29.24	27.68	14.63	42.30	57.70	–0.33	0.09
KF664133.1-KF664142.1	30.39	26.07	26.02	17.52	43.54	56.46	50.62	36.84	43.73	27.11	29.73	28.25	14.91	43.16	56.84	–0.34	0.09
KF664123.1-KF664132.1	30.62	25.82	25.70	17.86	43.56	56.44	50.73	37.05	43.89	27.79	29.33	27.48	15.41	42.88	57.12	–0.35	0.09
KF664113.1-KF664122.1	30.74	25.89	25.55	17.82	43.37	56.63	50.49	37.13	43.81	28.03	29.48	27.04	15.46	42.49	57.51	–0.35	0.09
KF664103.1-KF664112.1	30.42	26.02	25.99	17.57	43.56	56.44	50.59	36.85	43.72	27.13	29.64	28.20	15.04	43.24	56.76	–0.34	0.09
KJ019205.1-KJ019214.1	30.28	25.82	26.32	17.58	43.90	56.10	51.13	37.17	44.15	27.11	29.50	28.85	14.55	43.39	56.61	–0.34	0.09
KJ577094.1-KJ577103.1	30.28	25.82	26.32	17.58	43.90	56.10	51.12	37.17	44.14	27.11	29.48	28.85	14.56	43.41	56.59	–0.34	0.09
KF560417.1-KF560426.1	30.25	25.93	26.03	17.79	43.82	56.18	50.69	37.08	43.89	26.88	29.44	28.17	15.52	43.68	56.32	–0.33	0.09
KJ577104.1-KJ577113.1	30.37	25.78	26.25	17.60	43.85	56.15	51.05	37.16	44.10	27.33	29.34	28.64	14.69	43.33	56.67	–0.34	0.09
KJ577114.1-KJ577123.1	30.33	25.78	26.30	17.59	43.89	56.11	51.00	37.19	44.10	27.15	29.35	28.85	14.64	43.49	56.51	–0.34	0.09
KP339244.1-KP339253.1	30.40	25.94	25.90	17.75	43.65	56.35	50.74	37.08	43.91	27.52	29.34	27.88	15.25	43.14	56.86	–0.33	0.09
KP339184.1-KP339193.1	30.38	25.94	25.98	17.70	43.68	56.32	50.37	37.24	43.80	27.12	29.44	28.23	15.20	43.44	56.56	–0.35	0.09
KP339174.1-KP339183.1	30.39	25.94	25.96	17.70	43.67	56.33	50.36	37.21	43.78	27.12	29.44	28.22	15.21	43.43	56.57	–0.35	0.09
KP339214.1-KP339223.1	30.45	25.88	26.17	17.50	43.67	56.33	50.58	36.88	43.73	26.94	29.49	28.46	15.11	43.57	56.43	–0.35	0.09
KP339204.1-KP339213.1	30.38	26.02	26.00	17.60	43.60	56.40	50.58	36.85	43.72	26.98	29.65	28.25	15.13	43.37	56.63	–0.35	0.09
KP339194.1-KP339203.1	30.39	26.03	25.98	17.60	43.58	56.42	50.57	36.85	43.71	27.02	29.66	28.16	15.16	43.32	56.68	–0.35	0.09
KP339144.1-KP339153.1	30.58	25.77	25.77	17.88	43.65	56.35	50.75	37.13	43.94	27.69	29.23	27.59	15.49	43.08	56.92	–0.35	0.09
KP339134.1-KP339143.1	30.63	25.86	25.72	17.79	43.51	56.49	50.76	37.06	43.91	27.84	29.45	27.46	15.25	42.71	57.29	–0.35	0.09
KC662612.1-KC662621.1	30.49	25.73	26.19	17.59	43.78	56.22	50.91	37.55	44.23	27.51	29.62	27.89	14.98	42.87	57.13	–0.35	0.09
KX164039.1-KX164048.1	30.52	25.84	25.90	17.73	43.64	56.36	50.82	36.96	43.89	28.04	28.83	27.83	15.30	43.13	56.87	–0.34	0.09
KX164029.1-KX164038.1	30.47	25.63	25.96	17.94	43.90	56.10	50.68	36.92	43.80	27.57	28.32	28.25	15.85	44.11	55.89	–0.35	0.09
KX164019.1-KX164028.1	30.25	25.76	26.46	17.53	43.99	56.01	50.84	37.27	44.05	27.12	29.03	29.09	14.76	43.85	56.15	–0.33	0.09
KT885075.1-KT885084.1	30.34	25.68	26.00	17.98	43.98	56.02	51.12	37.52	44.32	27.67	29.02	27.77	15.54	43.31	56.69	–0.33	0.09
KT885065.1-KT885074.1	30.37	25.54	26.06	18.03	44.09	55.91	50.90	37.00	43.95	27.15	28.49	28.40	15.96	44.37	55.63	–0.33	0.09
KT885055.1-KT885064.1	30.34	25.69	26.00	17.98	43.98	56.02	51.13	37.49	44.31	27.67	29.02	27.77	15.54	43.31	56.69	–0.33	0.09
KJ736001.1-KJ736010.1	30.28	25.74	26.33	17.64	43.98	56.02	51.14	37.19	44.17	27.09	29.31	28.84	14.76	43.60	56.40	–0.34	0.09
KX164139.1-KX164148.1	30.45	25.66	26.40	17.49	43.90	56.10	50.59	37.48	44.03	27.53	28.85	28.64	14.99	43.62	56.38	–0.34	0.09
KX164119.1-KX164128.1	30.29	25.70	26.30	17.70	44.00	56.00	50.59	37.36	43.98	27.01	28.93	28.70	15.35	44.06	55.94	–0.33	0.09
KX164059.1-KX164068.1	30.33	25.97	26.21	17.49	43.70	56.30	50.71	37.31	44.01	27.57	29.33	28.49	14.61	43.09	56.91	–0.33	0.09

Range	30.19	25.43	25.55	16.99	43.29	55.73	50.18	36.82	43.61	26.74	28.22	27.04	13.55	41.70	55.47	–0.35	0.09
	30.75	26.21	26.48	18.03	44.27	56.71	51.14	37.60	44.32	28.46	29.94	29.09	15.96	44.53	58.30	–0.31	0.09
Mean ±	30.42	25.86	26.07	17.65	43.71	56.29	50.69	37.17	43.93	27.45	29.27	28.21	15.07	43.28	56.72	–0.34	0.09
STD	0.14	0.15	0.23	0.20	0.21	0.21	0.23	0.22	0.17	0.43	0.37	0.47	0.47	0.51	0.51	0.01	0.00

*GC12 represents the G + C content at the first and second positions of codons. GC3 represents the G + C content at the third positions of codons. AU3 represents the A + U content at the third positions of codons. Gravy represents the hydrophobicity of protein. ARO represents the aromaticity of protein.*

### Relative Aynonymous Codon Usage (RSCU) Analysis

To understand the reason why A/U nucleotides were preferred at the third codon position, RSCU analysis was performed to describe the codon usage bias of BTV. The RSCU values of all synonymous codons were calculated for 50 BTV strains and compared with those of their hosts (Table 2). The result showed that there are 14 codons (UUU, UUA, AUU, GUU, UCA, CCA, UAU, CAU, CAA, AAU, GAU, UGU, AGA, and GGA) that are A/U-ended (A-ended: 6; U-ended: 8) among the 18 abundant codons in BTVs, while the remaining four (ACG, GCG, AAG, and GAG) are G/C-endings. Accordingly, this result is consistent with earlier studies that A/U-ended codons have increased abundance in the virus genome, such as Crimean–Congo hemorrhagic fever virus, avian rotaviruses, and equine influenza viruses (Kattoor et al., 2015; Kumar et al., 2016; Rahman et al., 2018). Analysis of over- and underrepresented codons emphasized that the RSCU values of the majority of codons ranged from 0.6 to 1.6. Remarkably, the results also showed that a majority of overpreferred codons (RSCU > 1.6) had A-endings, while the most underpreferred codons (RSCU < 0.6) had G-endings (Table 2), showing that mutational bias was the driving force for codon usage patterns in BTV. In addition, to evaluate whether the codon usage bias of BTV can be limited by its vector and hosts (including *Culicoides*, *B. taurus*, and *O. aries*), the RSCU values of all codons in them were also calculated (Table 2). This analysis suggested that 9 and 16 of 59 synonymous codons of BTV are similar to those of *B. taurus*, or *O. aries* individually, and that 24 of 59 synonymous codons are similar to those of the vector (*Culicoides*) (Table 2). It was suggested that the similarity of codon usage patterns between BTVs and their hosts can improve the translation efficiency of viral genomes.

**TABLE 2:** Comparison of RSCU value of different codons of BTV and its host (*B. taurus*, *O. aries*, *Culicoides*).

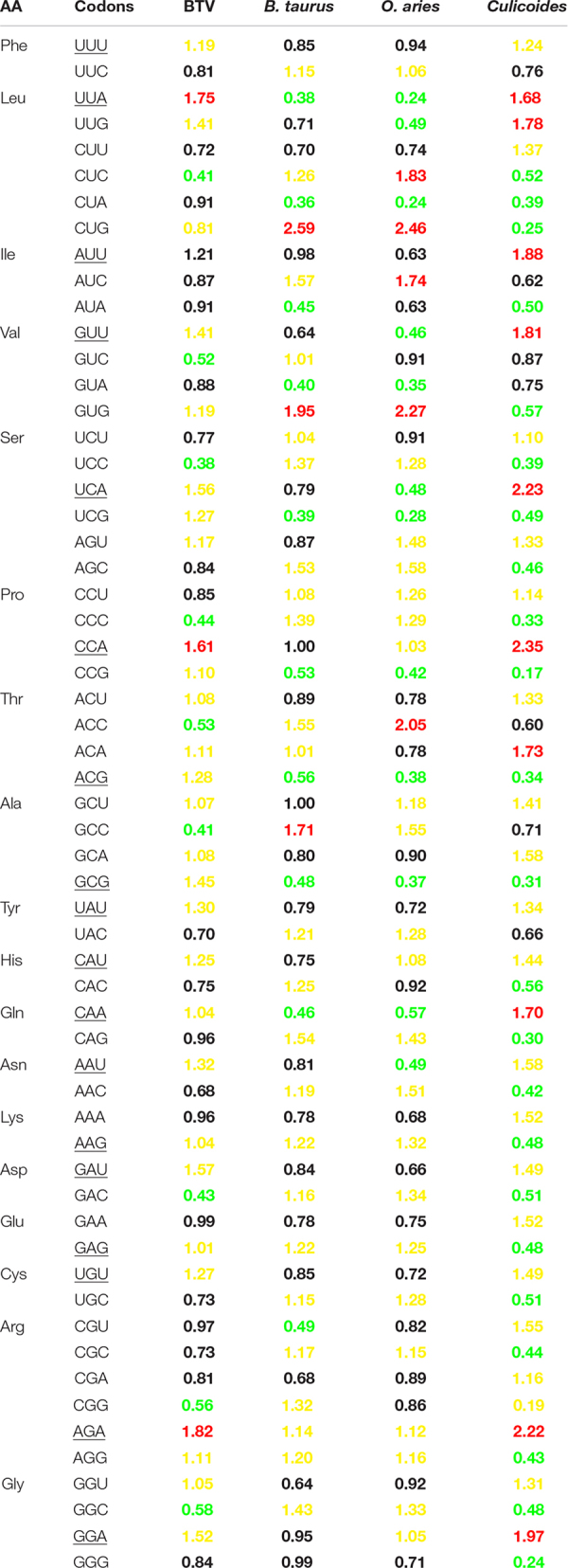

*AA, amino acid; RSCU, relative synonymous codon usage value. Orange colors denote codons favored by BTV and hosts (RSCU > 1). Overrepresented (RSCU > 1.6) and underrepresented (RSCU < 0.6) codons are marked as bold with red and green colors, respectively. The optimal codons for BTV are underlined.*

### BTV Codon Usage Is Largely Shaped by Mutation Pressure

We continuously calculated the ENC to evaluate the magnitude of codon usage bias among all BTV coding sequences. The range of the ENC value is between 20 and 61, and the lower the ENC value is, the stronger the preference of codon usage (Wright, 1990; Comeron and Aguadé, 1998). ENC is acquired by considering the contributions of each of the five synonymous family types. Preliminary results showed that the ENC values ranged from 53.62 to 55.63 (mean = 54.90, SD = 0.40) (Supplementary Table S1), which were higher than 35, showing equally and slightly biased codon usage of all BTV genomes.

**FIGURE 2:**
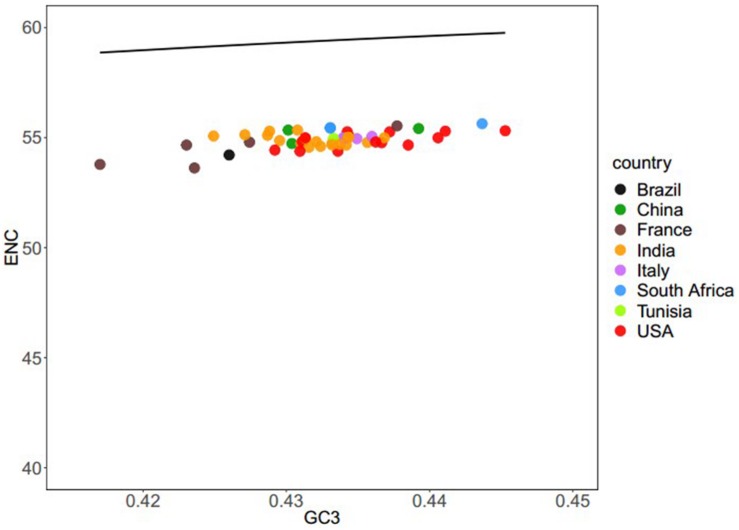
ENC–GC3 plots of 50 BTV strains: different BTV strains are shown in various color schemes. The solid line indicates the expected ENC vs. GC3 plot under the null model. We explored codon usage for all BTV strains. The plots concentrated between the 53.62 and 55.63 range suggest that codon usage bias is caused by mutational pressure.

To evaluate the degree of codon usage patterns among the coding sequences of all the different BTV isolates, an ENC-GC3 plot was produced. This plot is used to determine whether the codon usage pattern of a gene deviates from the equivalent usage of the corresponding synonymous codons (Wright, 1990; Wu et al., 2015). If there is no natural selection, genetic evolution is affected only by mutation pressure. The nucleotide composition of the genome sequence would be the only way to affect its codon usage bias. Therefore, each point will fall on the expected curve or near the expected curve. Conversely, if the points are below the expected curve, the gene expression is subject to natural selection. As Figure 2 shows, all the points lie greatly below the solid curve, which suggests that in addition to the mutation pressure, translation selection also influences the codon usage bias of BTV. Our results are consistent with previous studies (Butt et al., 2014; Chen H. et al., 2014; Wang et al., 2016; Rahman et al., 2018).

To estimate the contribution of mutation bias and natural selection, a neutrality plot was produced for GC12 and GC3 contents. In the plot, the regression coefficient against GC3 is regarded as the mutation–selection equilibrium coefficient and the evolutionary speed of the mutation pressure and natural selection pressure is expressed as the slope of a regression line. Each point represents a species corresponding to the composition of GC12 and GC3 from the neutrality plot. However, if all the points lie along the diagonal distribution, no significant difference exists at the three codon positions, and there is no or weak external selection pressure. Alternatively, if the regression curve tends to be sloped or parallel to the horizontal axis, then the variation correlation between GC12 and GC3 is very low. The result showed that the correlation between GC12 and GC3 is not significant (*r* = 0.09, *P* > 0.05), reflecting that both mutation pressure and natural selection shape the codon usage pattern of BTV (Supplementary Figure S1).

### The Variation in Codon Usage Among all BTVs

Principal component analysis (PCA) is used to explore the variation in codon usage based on the RSCU values of all BTV isolates. Here, the first two principal components from the PCA were determined to offer two-dimensional visualization of the sample relationships. The results identified that the first principal component accounted for 69.91% and the second principal component accounted for 27.95% of the variance (Supplementary Figure S2). Scattered points in the plot describe the diverse geographical lineages and their connection with each other.

**FIGURE 3:**
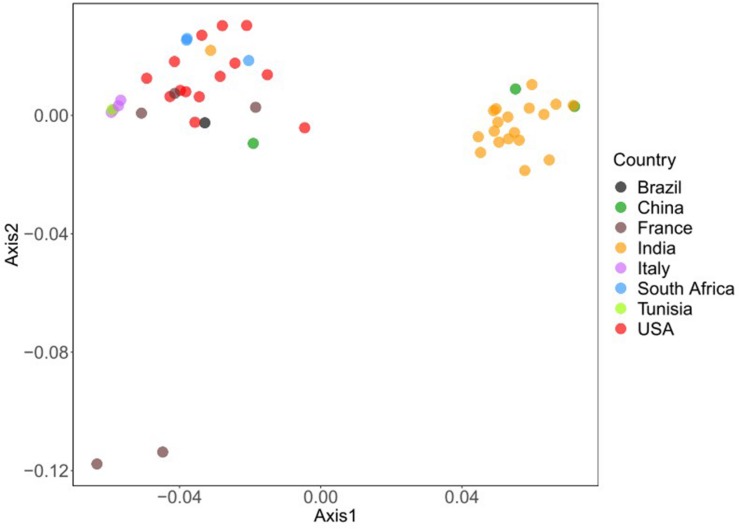
Variation analysis in BTV genomes: based on the RSCU values, all the strains are plotted in variance plane. The first and second principal axes represent different geographical origin. Each point represents a strain and shows in different colors.

Correspondence analysis was also used on RSCU values of all viral sequences to visualize and explore these data. For large multidimensional variables, COA can reduce the dimensions of the datasets to achieve efficient visualization of numerous variables (Mallows et al., 1985). The results displayed that all BTV strains were collected into clusters (Figure 3). All BTV strains from the United States, Italy, France, and Brazil are assembled in one cluster, while BTV strains from India are grouped in another cluster. However, some BTV strains from China appeared in the different clusters. These results suggested that the geographical locations play an important role in BTV evolutionary process and a synonymous codon usage pattern. Besides, it was also highlighted that each infected country has emerged more than one viral genetic lineage.

### Codon Usage Adaptation in BTVs

**FIGURE 4:**
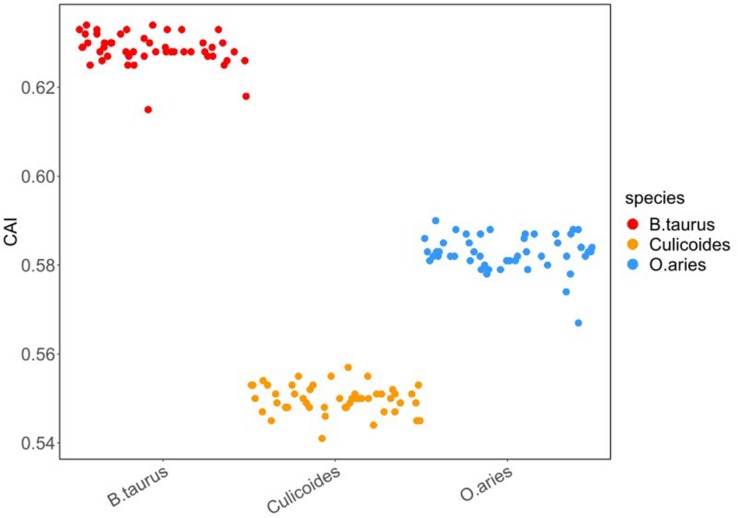
CAI of BTV to its hosts. In the plot, the *x*-axis represents the vector (*Culicoides*) and the host species (*B. taurus* and *O. aries*). The *y*-axis represents the CAI value. Different colors represent various species: red—*B. taurus*, blue—*O. aries*, and orange—*Culicoides*. To avoid the CAI value between the BTV and each host overlapping, we performed a 50% disturbance in the horizontal direction (width = 0.5, height = 0).

To investigate the optimal codon usage pattern of BTVs and the adaptation in their hosts, the codon adaptation index (CAI) of all strains was measured by taking the codon usage patterns of *B. taurus*, *O. aries*, and *Culicoides* as a reference. The range of CAI values is between 0 and 1; the higher CAI values, the better adaptation of virus (Butt et al., 2014). In our research, the CAI values of all the BTV isolates were 0.63 ± 0.004, 0.58 ± 0.004, and 0.55 ± 0.003 in reference to *B. taurus*, *O. aries*, and *Culicoides* codon usage patterns, respectively (Figure 4). Furthermore, the significant differences determined in this study were calculated by Mann–Whitney *U* test, and it was shown that there were significant differences among CAI values (Supplementary Table S2, wilcox.test, *P* < 0.01). In addition, we calculated the CAI values of BTV in relation to itself and suggested that BTVs were better adapted to their hosts (*B. taurus* and *O. aries*) than to their vector (*Culicoides*) (Supplementary Table S2).

The expected CAI (e-CAI) values were also obtained for the whole BTV strains in reference to *B. taurus, O. aries*, and *Culicoides* to discern whether the differences in the CAI value were statistically significant (Puigbo et al., 2008). The e-CAI values of 0.68 (*P* < 0.05), 0.64 (*P* < 0.05), and 0.61 (*P* < 0.05) for *B. taurus*, *O. aries*, and *Culicoides*, respectively, suggested that there was a normal distribution of all the generated sequences. Figure 4 shows that the CAI values for BTVs in relation to *Culicoides* are significantly different from those in relation to *B. taurus* and *O. aries* (wilcox.test, *P* < 0.01). The result reflects that the selection pressure from hosts may influence the codon usage pattern of BTV and that the translation resources of hosts are more efficient than those of the vector for BTV.

### The Main Constraints of the Codon Usage Pattern

In view of two constraints (including mutation pressure and natural selection) of codon usage patterns in BTV, we further analyzed the correlation between the ENC and CAI values to examine the predominant factor. If the correlation coefficient (*r*) between two indices is close to 1, translational selection is the primary determinant, whereas the mutation pressure may be more preferred than translational selection (Wang et al., 2016). There were significant correlations between ENC and CAI values of BTV coding sequences in reference to *B. taurus* (*r* = 0.43, *P* < 0.01), *O. aries* (*r* = 0.52, *P* < 0.01), and *Culicoides* (*r* = −0.32, 0.01 < *P* < 0.05), indicating that the codon usage pattern of BTV genomes is limited by both natural selection and mutational pressure (Table 3). Furthermore, correlation analysis among T (−0.66), C^3^ (0.63), and GC (0.51) with ENC was also performed by Spearman’s rank correlation (Table 4).

**TABLE 3:** The correlation between CAI and ENC.

	CAI (*O. aries*)	CAI (*B. taurus*)	CAI (*Culicoides*)
ENC	0.52**	0.43**	−0.32*

The numbers in each column represent correlation coefficient “*r*” values, which are calculated in each correlation analysis. NS, non-significant (*P* > 0.05). *0.01 < *P* < 0.05. **P < 0.01.

**TABLE 4:** Correlation analysis among GC, GC3s, GRAVY, ARO, ENC, and the first two principal axes of COA.

	GC	GC3	ENC	Gravy	ARO	Axis 1	Axis 2
**GC**		0.99**	0.51**	0.25^NS^	−0.35*	−0.36**	0.42**
**GC3**			0.52**	0.25^NS^	−0.35*	−0.38**	0.43*
**ENC**				−0.19^NS^	−0.05^NS^	0.14^NS^	0.46**
**Gravy**					−0.11^NS^	−0.60**	0.27^NS^
**ARO**						0.26^NS^	−0.16^NS^
**Axis1**							−0.23*

The numbers in each column represent correlation coefficient “*r*” values, which are calculated in each correlation analysis. NS, non-significant (*P* > 0.05). *0.01 < *P* < 0.05. **P < 0.01. Gravy represents the hydrophobicity of protein. ARO represents the aromaticity of protein.

**FIGURE 5:**
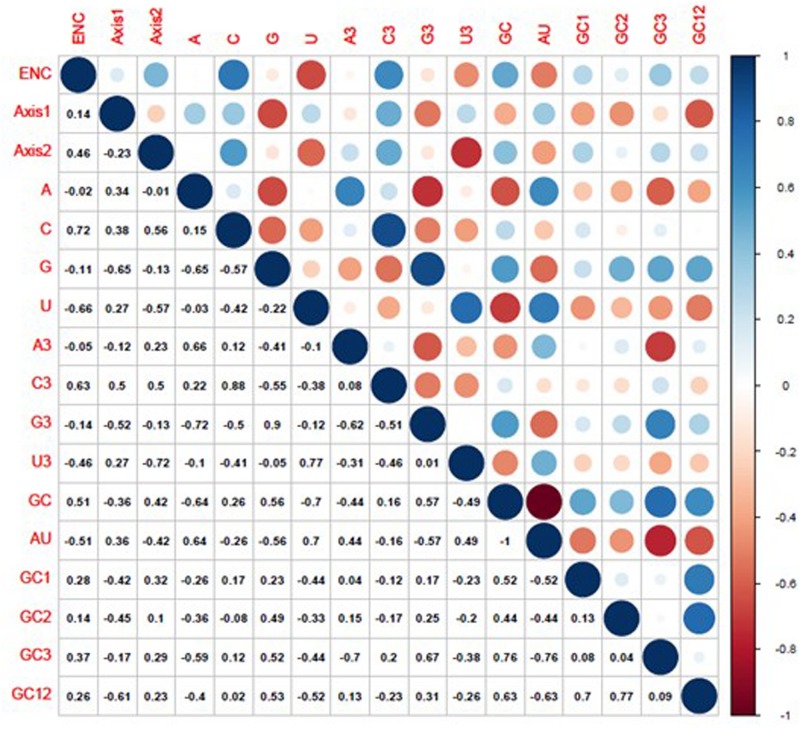
Correlation analysis among different indices of BTVs. The dark blue indicates a negative correlation, and the dark red indicates a positive correlation; the higher value indicates a more significant correlation.

The composition of the overall nucleotide and the third codon position nucleotide was used as a reference to evaluate the influence of nucleotide composition on the BTV codon usage pattern. It was demonstrated that GC3 (*r* = 0.76, *P* < 0.01), G3 (*r* = 0.9, *P* < 0.01), C3 (*r* = 0.88, *P* < 0.01), A3 (*r* = 0.66, *P* < 0.01), and U3 (*r* = 0.77, *P* < 0.01) have significant positive correlations with the set of full-length gene sequences (GC, G, C, A, and U) (Figure 5). The results above suggest that natural selection and nucleotide content influence BTV codon usage patterns. We further performed Spearman’s rank correlation analysis between the base contents of BTV and the two principal components (axis 1 and axis 2) (Supplementary Figure S2). Figure 5 indicates that there are significant correlations among nucleotide contents and the two principal components. The first axis shows a significant association with *G* (*r* = −0.65, *P* < 0.001), C^3^ (*r* = 0.50, *P* < 0.001), G^3^ (*r* = −0.52, *P* < 0.001), and GC^12^ (*r* = −0.61, *P* < 0.001), while the second axis correlates significantly with ENC (*r* = 0.46, *P* < 0.001), C (*r* = 0.56, *P* < 0.001), U (*r* = −0.57, *P* < 0.001), C3 (*r* = 0.50, *P* < 0.001), and U3 (*r* = −0.72, *P* < 0.001). These results validate that in addition to natural selection, nucleotide contents can also play a role in synonymous codon usage patterns.

## Discussion

This survey of the BTV complete genomes indicates a preference for A/U nucleotide over G/C nucleotide and that impacts the codon usage for translation of viral proteins. This finding is similar to previous research on Crimean–Congo hemorrhagic fever virus (CCHFV) being enriched with A and U (Rahman et al., 2018). However, the biological significance of this condition is still unclear, and therefore it is vital to explore the causes for significantly increased A content and concomitant decreased C content in the viral genomes (van Hemert and Berkhout, 2016). Some previous reports showed that the composition of amino acids was also the key factor in determining the nucleotide contents at the first and second codon positions of viral genomes, while the variation in proteins was forced by functional selection. However, 69% of the alteration at the third codon position always denoted synonymous or silent mutations, which was not affected by functional selection of protein products (van Hemert and Berkhout, 2016).

Earlier research suggested that the codon usage pattern of Ebola virus (EBOV) was different from that of its hosts (Schubert and Catherine, 2010; Cristina et al., 2015). Our results are consistent with earlier studies showing that A/U-ended codons are more abundant in the BTV genome than in the host genome (Rabadan et al., 2006; Greenbaum et al., 2008). In addition, some previous reports also indicated that the identical compositions of codon usage patterns between viruses and hosts could increase the translation efficiency of the corresponding amino acids, while the contrary compositions of codon usage patterns could ensure the correct folding of viral proteins (Aragones et al., 2010; Costafreda et al., 2014; Hu et al., 2014; Cristina et al., 2015). These results also reflect that the similar usage of codons between BTV and its common hosts may enhance the ability of viral genes to participate in the translation process. Specifically, the codon usage pattern of BTV genomes may be largely influenced by the selection pressure of its natural hosts, which can be conducive to adaptation to the cellular conditions of its hosts and efficient replication (Wong et al., 2010; Ma et al., 2015). However, the influence of selection from hosts (*B. taurus*, *O. aries*) on shaping codon usage patterns of BTV is not similar to the vector (*Culicoides*). Previous researches on EBOV and *Flaviviridae* virus suggested that the codon usage patterns are very different with their hosts (Schubert and Catherine, 2010; Cristina et al., 2015).

In this study, a number of systemic analytical approaches were performed to explore the factors shaping the BTV codon usage patterns. To start with, an ENC–GC3 analysis was performed. In BTV genomes, the ENC values were considered to estimate the codon usage bias in the complete viral genomes. The result shows that overall codon usage bias of BTV genomes was low (ENC = 57.9). It has also been found among some other viruses, such as hepatitis C virus (ENC = 52.62) (Hu et al., 2011), Ebola virus (ENC = 57.23) (Cristina et al., 2015), and Crimean–Congo hemorrhagic fever virus (ENC = 52.34) (Rahman et al., 2018). It has been indicated that the low codon usage bias of virus is beneficial for the efficient replication in its host cells and the reduced competition between virus and its host for the protein synthesis. Although ENC values can estimate the codon usage bias of BTV genomes, these values alone cannot be used to reflect the driving force of codon usage bias. An ENC plot of BTV genomes whose codon usage patterns are only constrained by their GC3 compositions will lie on or slightly below the solid line of the expected ENC values. When present, this influence of nucleotide constraints indicates the fundamental effect of mutation pressure. In BTV genomes, we observed AU compositions to be significantly higher than GC compositions. To measure how this genomic content may have impacted the codon usage patterns of BTV, we derived our assumption from ENC–GC3 analysis. It shows that both natural selection and mutational pressure have affected the codon usage patterns in BTV complete genomes.

An earlier study also suggested that mutation pressure was the dominant factor affecting the codon usage pattern of *Zaire ebolavirus* (ZEBOV). Studies have shown that natural selection is largely limited by nucleotide contents in the first and second sites of codons, although the mutational pressure is generally limited by the nucleotide contents in the third site of codons (Roychoudhury and Mukherjee, 2010; Hu et al., 2014). Therefore, we further performed Spearman’s rank correlation analysis between the base contents of BTV and the two principal components (axis 1 and axis 2) and validated that in addition to natural selection, nucleotide contents can also play a role in synonymous codon usage patterns.

Except for natural selection and mutation pressure, other factors including geographic origins and translation selection can also influence the viral codon usage patterns (Chen Y. et al., 2014; Rahman et al., 2018). The results of the COA suggested that all BTV strains are collected into clusters (Figure 3). These results highlight that the geographical location of BTVs plays a significant role in their evolution and codon usage patterns. These results also indicate that there is more than one prevailing genetic lineage in each infected country and promote studies to trace the origin of the existing BTVs. Besides, the results of CAI analysis reflect that the selection pressure from hosts may influence the codon usage pattern of BTV and that the translation resources of hosts are more efficient for BTV than those of the vector. These results supported the role of geographical locations and translational selection on codon usage patterns of BTV genomes. In addition, our study also reveals that the differences among various hosts are associated with the codon usage bias of BTV. It is compatible with previous studies that have shown distinct codon usage patterns between virus and host genes (Schubert and Catherine, 2010; Cristina et al., 2015).

## Conclusion

According to the available evidence, our findings suggest that analysis of codon usage bias can offer an alternative strategy to explore the evolution of BTVs. Using PCA and COA on RSCU values, the codon usage patterns and trends of BTV strains were obtained. Furthermore, the results may distinguish the different viral groups and expose their evolutionary trends. Further studies of codon usage demonstrated that the evolution of BTV could be regulated mainly by natural selection in addition to mutation pressure. The observed nucleotide composition might also be the driving force shaping the codon usage patterns of BTVs. Additionally, it is suggested that there are similarities of codon usage between BTVs and their hosts. This research not only provides the knowledge about the variation in BTV codon usage patterns but also contributes to analyzing the factors that drive BTV evolution.

## Data Availability Statement

All datasets generated for this study are included in the article/Supplementary Material.

## Author Contributions

DC and ST conceived and designed the experiments. XY and BY performed all the experiments. XY, QF, and BY collected and analyzed the data. SR and PL drafted the manuscript. All authors read and approved the final manuscript.

## Conflict of Interest

The authors declare that the research was conducted in the absence of any commercial or financial relationships that could be construed as a potential conflict of interest.

## Footnotes

1 http://www.ncbi.nlm.nih.gov/GenBank

2 http://www.kazusa.or.jp/codon/

3 http://ppuigbo.me/programs/CAIcal/

4 http://sourceforge.net/projects/codonw

## Supplementary Material

FIGURE S1 Neutrality plot analysis (GC12 vs. GC3) for the entire coding sequences of BTVs. GC12 indicates the average value of GC contents at the first and second codon positions (GC1 and GC2), while GC3 refers to the GC contents at the third codon position. The red dotted line is the linear regression of GC12 against GC3, *R*^2^ = 0.001271, *P* > 0.05.

FIGURE S2 A plot of all BTV complete sequences in PCA. The first axis accounts for 69.91% of the total variation, and the second axis accounts for 27.95% of the total variation.

TABLE S1 The information of BTV strains in this study.

TABLE S2 Codon adaptation index (CAI) value.
