# Supplementary figures and images for: Codon Usage Bias Analysis of Bluetongue Virus Causing Livestock Infection
Source: Front Microbiol. 2020 May 19;11:655. doi: 10.3389/fmicb.2020.00655 (PMC7248248; doi:10.3389/fmicb.2020.00655)

Fig. S1

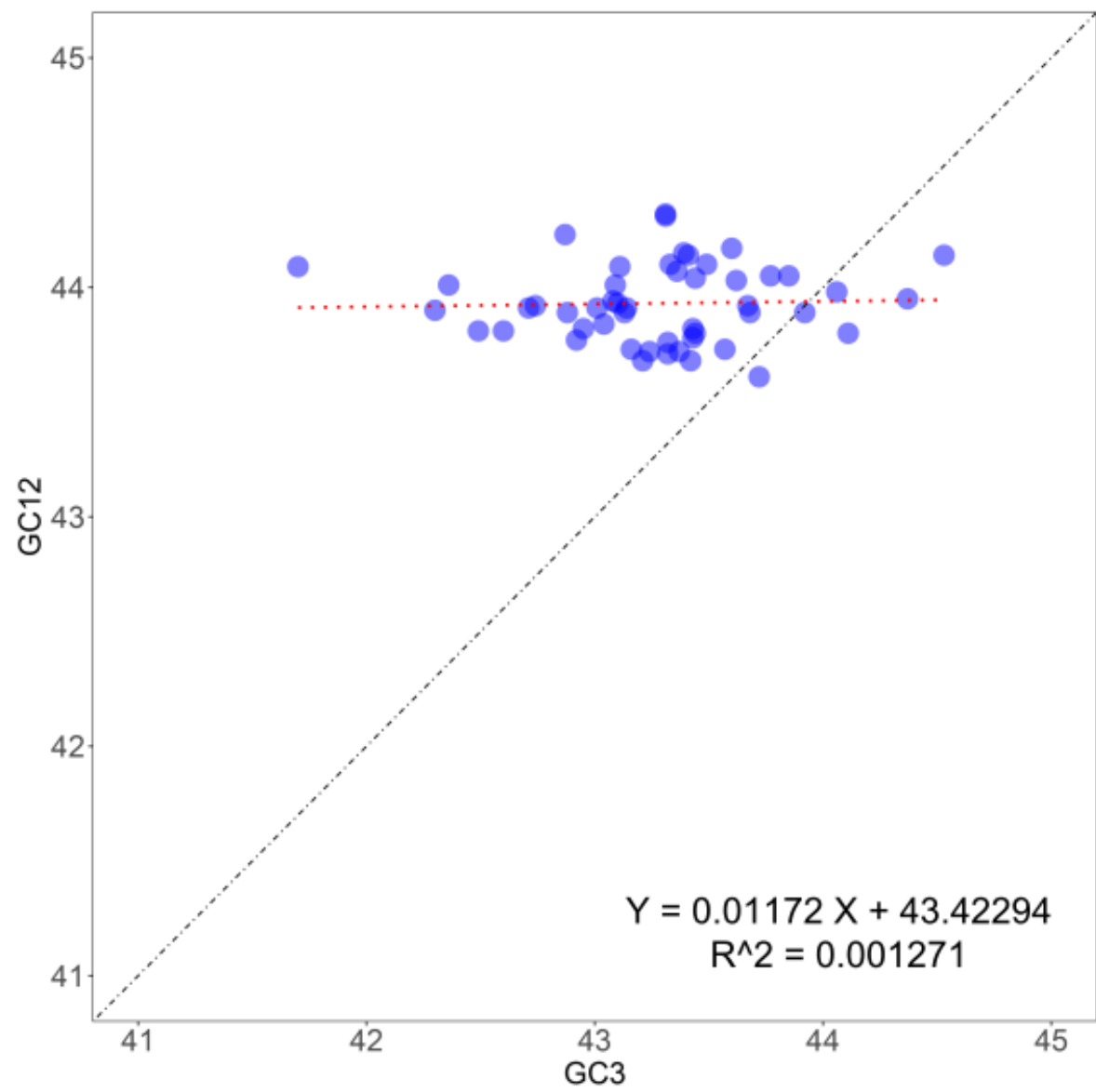

Fig. S2

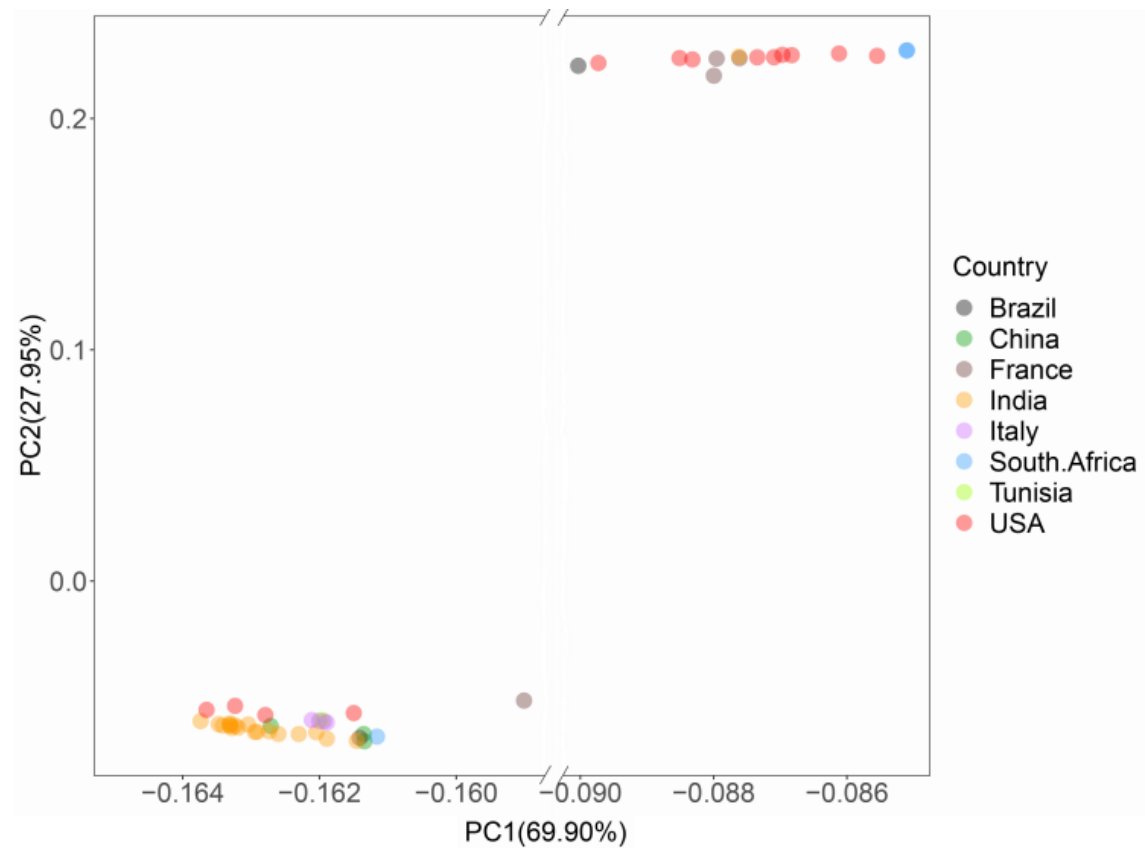

Supplement: FIGURE S1 — Neutrality plot analysis (GC12 vs. GC3) for the entire coding sequences of BTVs. GC12 indicates the average value of GC contents at the first and second codon positions (GC1 and GC2), while GC3 refers to the GC contents at the third codon position. The red dotted line is the linear regression of GC12 against GC3, R2 = 0.001271, P > 0.05. [file Data_Sheet_1.pdf]
