# Supplementary material for: Codon Usage Bias Analysis of Bluetongue Virus Causing Livestock Infection
Source: Front Microbiol. 2020 May 19;11:655. doi: 10.3389/fmicb.2020.00655 (PMC7248248; doi:10.3389/fmicb.2020.00655)
Supplement: TABLE S1 — The information of BTV strains in this study. [file Data_Sheet_2.pdf]

**Table S1:** The information of BTV strains in this study.

| No. | Accession no. | Country | Host | Isolation | ENC | %GC3 |
|---|---|---|---|---|---|---|
| 1 | MG206077.1-MG206086.1 | China | cattle | 5149E | 54.73 | 43.04 |
| 2 | KP339154.1-KP339163.1 | India | sheep | BTV02IND1993 | 54.86 | 42.95 |
| 3 | KP339234.1-KP339243.1 | India | sheep | KMN07/05 | 54.73 | 43.10 |
| 4 | KP339224.1-KP339233.1 | India | sheep | BTV16IND2010-VC07 | 54.66 | 43.42 |
| 5 | KP339164.1-KP339173.1 | India | sheep | BTV02IND2010-KRM08 | 54.75 | 43.32 |
| 6 | KX599359.1-KX599368.1 | Brazil | sheep | BRA73 | 54.21 | 42.60 |
| 7 | KX164149.1-KX164158.1 | USA | white-tailed deer | USA2007/FL 520518 | 55.26 | 43.72 |
| 8 | KX164129.1-KX164138.1 | USA | cattle | USA2003/FL 280559-3 | 54.38 | 43.36 |
| 9 | KX164109.1-KX164118.1 | USA | domestic sheep | USA2003/FL 279313 | 54.77 | 43.67 |
| 10 | KX164099.1-KX164108.1 | USA | pronghorn antelope | USA2013/CA 13-034210 | 55.26 | 43.43 |
| 11 | KX164089.1-KX164098.1 | USA | white-tailed deer | USA2012/LA 12-046093 | 55.09 | 43.44 |
| 12 | KX164079.1-KX164088.1 | USA | white-tailed deer | USA2008/TX 576181 | 55.31 | 44.53 |
| 13 | KX164069.1-KX164078.1 | USA | deer | USA2008/FL 576307 | 54.82 | 43.11 |
| 14 | KX164049.1-KX164058.1 | USA | cattle | USA2015/TX 15-029176 | 54.43 | 42.92 |
| 15 | JX003687.1-JX003696.1 | India | sheep | Ind-R1-2007 | 54.81 | 43.21 |
| 16 | KU760997.1-KU761006.1 | France | goat (Capra aegagrus hircus) | BTV-27/FRA2014/v03 | 53.78 | 41.70 |
| 17 | KU760987.1-KU760996.1 | France | goat (Capra aegagrus hircus) | BTV-27/FRA2014/v02 | 53.62 | 42.36 |
| 18 | KT002578.1-KT002587.1 | China | cattle | GDST008 | 55.41 | 43.92 |
| 19 | JX399148.1-JX399157.1 | China | sheep | K23/08 | 55.34 | 43.01 |
| 20 | KY654328.1-KY654337.1 | France | sheep | BTV-4/16-03 | 55.53 | 43.77 |
| 21 | KY049853.1-KY049862.1 | France | bovine | BTV-1/15.01 (5370) | 54.79 | 42.74 |
| 22 | KY049843.1-KY049852.1 | France | bovine | BTV-1/11-01 (4074) | 54.66 | 42.30 |
| 23 | KF664133.1-KF664142.1 | India | goat; breed: Mehsana | G53/ABT/HSR | 54.56 | 43.16 |
| 24 | KF664123.1-KF664132.1 | India | sheep | NRT37/ABT/HSR | 55.29 | 42.88 |
| 25 | KF664113.1-KF664122.1 | India | Culicoides oxystoma | SKN7/ABT/HSR | 55.07 | 42.49 |
| 26 | KF664103.1-KF664112.1 | India | sheep | K31-08/ABT/HSR | 54.60 | 43.24 |
| 27 | KJ019205.1-KJ019214.1 | Italy | sheep | BTV-1 SAD2013 | 54.98 | 43.39 |
| 28 | KJ577094.1-KJ577103.1 | Italy | sheep | SAD2012 | 54.98 | 43.41 |

| | | | | | | |
|---|---|---|---|---|---|---|
| 29 | KF560417.1-KF560426.1 | India | sheep | WGV103/ABT/HSR | 54.99 | 43.68 |
| 30 | KJ577104.1-KJ577113.1 | Tunisia | sheep | TUN2011 | 54.96 | 43.33 |
| 31 | KJ577114.1-KJ577123.1 | Italy | sheep | SAD2010 | 54.95 | 43.49 |
| 32 | KP339244.1-KP339253.1 | India | sheep | BTV10IND2003K3 | 54.95 | 43.14 |
| 33 | KP339184.1-KP339193.1 | India | sheep | BTV09IND2003-M11 | 55.01 | 43.44 |
| 34 | KP339174.1-KP339183.1 | India | sheep | BTV09IND2003-M10 | 55.02 | 43.43 |
| 35 | KP339214.1-KP339223.1 | India | sheep | BTV16IND2011-NR82 | 54.78 | 43.57 |
| 36 | KP339204.1-KP339213.1 | India | sheep | BTV16IND2010-AP06 | 54.69 | 43.37 |
| 37 | KP339194.1-KP339203.1 | India | sheep | BTV16IND2010-AP04 | 54.67 | 43.32 |
| 38 | KP339144.1-KP339153.1 | India | sheep | BTV01IND2010-VC12 | 55.34 | 43.08 |
| 39 | KP339134.1-KP339143.1 | India | sheep | BTV01IND2010-KRM07 | 55.13 | 42.71 |
| 40 | KC662612.1-KC662621.1 | India | sheep | INDAPADBNMO1/11 | 55.10 | 42.87 |
| 41 | KX164039.1-KX164048.1 | USA | deer | USA2012/SD 12-035694 | 54.99 | 43.13 |
| 42 | KX164029.1-KX164038.1 | USA | white-tailed deer | USA2008/AR 566195 | 55.29 | 44.11 |
| 43 | KX164019.1-KX164028.1 | USA | domestic sheep | USA2010/FL 10-044273 | 54.66 | 43.85 |
| 44 | KT885075.1-KT885084.1 | South Africa | Bos taurus | B.taurus-tc/ZAF/2014/Onderstepoort-BTV-9_buffycoat_Bos_taurus_plaque2B | 55.43 | 43.31 |
| 45 | KT885065.1-KT885074.1 | South Africa | Ovis aries | O.aries-vaccine/ZAF/2014/Onderstepoort- OBPvaccine-batch115-expiry11102015-MLV11 | 55.63 | 44.37 |
| 46 | KT885055.1-KT885064.1 | South Africa | Ovis aries | O.aries-vaccine/ZAF/2014/Onderstepoort-OBP_vaccinebottleB_batch115 | 55.45 | 43.31 |
| 47 | KJ736001.1-KJ736010.1 | Italy | none | SAD2006 | 55.05 | 43.60 |
| 48 | KX164139.1-KX164148.1 | USA | domestic sheep | USA2005/FL 402286 | 54.80 | 43.62 |
| 49 | KX164119.1-KX164128.1 | USA | white-tailed deer | USA2014/FL 15-008010 | 54.99 | 44.06 |
| 50 | KX164059.1-KX164068.1 | USA | cattle | USA2003/FL 280559-7 | 54.38 | 43.09 |

| | | | | | | |
|---|---|---|---|---|---|---|
| Mean ± STD | | | | | 54.90 ±0.40 | 43.28 ±0.51 |

**Table S2:** Codon adaptation index (CAI) Value

| Accession No. | CAI(*O.aries*) | CAI(*B.taurus*) | CAI(*Culicoides*) | CAI(BTV) |
|---|---|---|---|---|
| MG206077.1-MG206086.1 | 0.579 | 0.626 | 0.553 | 0.579 |
| KP339154.1-KP339163.1 | 0.581 | 0.628 | 0.555 | 0.580 |
| KP339234.1-KP339243.1 | 0.578 | 0.625 | 0.551 | 0.576 |
| KP339224.1-KP339233.1 | 0.581 | 0.628 | 0.55 | 0.574 |
| KP339164.1-KP339173.1 | 0.583 | 0.629 | 0.553 | 0.577 |
| KX599359.1-KX599368.1 | 0.582 | 0.626 | 0.557 | 0.579 |
| KX164149.1-KX164158.1 | 0.587 | 0.633 | 0.548 | 0.585 |
| KX164129.1-KX164138.1 | 0.581 | 0.628 | 0.549 | 0.561 |
| KX164109.1-KX164118.1 | 0.583 | 0.628 | 0.546 | 0.579 |
| KX164099.1-KX164108.1 | 0.587 | 0.633 | 0.55 | 0.575 |
| KX164089.1-KX164098.1 | 0.585 | 0.629 | 0.549 | 0.586 |
| KX164079.1-KX164088.1 | 0.586 | 0.63 | 0.541 | 0.579 |
| KX164069.1-KX164078.1 | 0.582 | 0.628 | 0.549 | 0.576 |
| KX164049.1-KX164058.1 | 0.586 | 0.63 | 0.554 | 0.570 |
| JX003687.1-JX003696.1 | 0.582 | 0.629 | 0.553 | 0.584 |
| KU760997.1-KU761006.1 | 0.567 | 0.615 | 0.55 | 0.569 |
| KU760987.1-KU760996.1 | 0.574 | 0.618 | 0.547 | 0.574 |
| KT002578.1-KT002587.1 | 0.585 | 0.631 | 0.547 | 0.602 |

| | | | | |
|---|---|---|---|---|
| JX399148.1-JX399157.1 | 0.582 | 0.627 | 0.55 | 0.589 |
| KY654328.1-KY654337.1 | 0.588 | 0.633 | 0.545 | 0.598 |
| KY049853.1-KY049862.1 | 0.585 | 0.63 | 0.553 | 0.582 |
| KY049843.1-KY049852.1 | 0.579 | 0.625 | 0.555 | 0.578 |
| KF664133.1-KF664142.1 | 0.579 | 0.627 | 0.552 | 0.573 |
| KF664123.1-KF664132.1 | 0.583 | 0.627 | 0.552 | 0.587 |
| KF664113.1-KF664122.1 | 0.582 | 0.627 | 0.555 | 0.584 |
| KF664103.1-KF664112.1 | 0.579 | 0.627 | 0.551 | 0.574 |
| KJ019205.1-KJ019214.1 | 0.587 | 0.632 | 0.548 | 0.600 |
| KJ577094.1-KJ577103.1 | 0.587 | 0.632 | 0.548 | 0.601 |
| KF560417.1-KF560426.1 | 0.583 | 0.629 | 0.55 | 0.577 |
| KJ577104.1-KJ577113.1 | 0.588 | 0.633 | 0.55 | 0.600 |
| KJ577114.1-KJ577123.1 | 0.587 | 0.632 | 0.548 | 0.597 |
| KP339244.1-KP339253.1 | 0.582 | 0.629 | 0.551 | 0.577 |
| KP339184.1-KP339193.1 | 0.583 | 0.63 | 0.551 | 0.587 |
| KP339174.1-KP339183.1 | 0.583 | 0.63 | 0.551 | 0.586 |
| KP339214.1-KP339223.1 | 0.584 | 0.63 | 0.548 | 0.576 |
| KP339204.1-KP339213.1 | 0.581 | 0.628 | 0.55 | 0.571 |
| KP339194.1-KP339203.1 | 0.581 | 0.628 | 0.551 | 0.571 |
| KP339144.1-KP339153.1 | 0.583 | 0.628 | 0.55 | 0.587 |
| KP339134.1-KP339143.1 | 0.58 | 0.625 | 0.553 | 0.581 |

| | | | | |
|---|---|---|---|---|
| KC662612.1-KC662621.1 | 0.578 | 0.625 | 0.553 | 0.583 |
| KX164039.1-KX164048.1 | 0.582 | 0.628 | 0.55 | 0.578 |
| KX164029.1-KX164038.1 | 0.588 | 0.634 | 0.547 | 0.581 |
| KX164019.1-KX164028.1 | 0.584 | 0.63 | 0.545 | 0.583 |
| KT885075.1-KT885084.1 | 0.582 | 0.628 | 0.55 | 0.572 |
| KT885065.1-KT885074.1 | 0.59 | 0.634 | 0.545 | 0.590 |
| KT885055.1-KT885064.1 | 0.582 | 0.628 | 0.549 | 0.572 |
| KJ736001.1-KJ736010.1 | 0.588 | 0.633 | 0.548 | 0.598 |
| KX164139.1-KX164148.1 | 0.584 | 0.63 | 0.549 | 0.586 |
| KX164119.1-KX164128.1 | 0.587 | 0.633 | 0.544 | 0.577 |
| KX164059.1-KX164068.1 | 0.58 | 0.626 | 0.551 | 0.574 |
| Mean± | 0.583 | 0.629 | 0.55 | 0.582 |
| STD | 0.004 | 0.004 | 0.003 | 0.009 |
